# Zebra finch males compensate in plumage ornaments at sexual maturation for a bad start in life

**DOI:** 10.1186/1742-9994-12-S1-S11

**Published:** 2015-08-24

**Authors:** E Tobias Krause, Marc Naguib

**Affiliations:** 1 Department of Animal Behaviour, University Bielefeld, Konsequenz 45, 33615 Bielefeld, Germany; 2 Institute of Animal Welfare and Husbandry, Friedrich-Loeffler Institute, Doernbergstr. 25-27, 29223 Celle, Germany; 3 Behavioural Ecology Group, Department of Animal Sciences, Wageningen University, De Elst 1, 6708 WD Wageningen, The Netherlands

**Keywords:** early developmental stress, fitness, personality, sexual selection, ornaments, nutritional stress, compensation, exploration behaviour

## Abstract

**Background:**

An individual's fitness in part depends on the characteristics of the mate so that sexually attractive ornaments, as signals of quality, are used in mate choice. Often such ornaments develop already early in life and thus are affected by nutritional conditions experienced then. Individuals thus should benefit by compensating as soon as possible for poor initial development of ornaments, to be attractive already at sexual maturity. Here, we tested whether early nutritional stress affects the cheek patch size of male Zebra finches (*Taeniopygia guttata*), which are important in mate choice, and whether a small cheek patch size early on is compensated at sexual maturation. Furthermore we tested whether exploration behaviour is affected by such a compensation, as shown for other compensatory growth trajectories.

**Results:**

Zebra finch males which were raised under poorer nutritional conditions initially expressed smaller cheek patches at day 50 post-hatching but then compensated in cheek patch size already at 65 days, i.e. when becoming sexually mature. Furthermore, compensatory growth in cheek patch during adolescence was negatively correlated with activity and exploration behaviour, measured in a novel environment.

**Conclusion:**

This compensation in cheek patch size benefits male attractiveness but also was related to less exploration behaviour, an established proxy for avian personality traits. We discuss the possibility that compensatory priorities exist so that not all deficits from a bad start are caught-up at the same time. Resource allocation to compensate for poorly expressed traits is likely to have evolved to optimise traits by the time they are most beneficial.

## Introduction

An individual's fitness is determined by its reproductive success relative to that of other individuals [1]. Part of this reproductive success will be determined by phenotypic characteristics such as those affecting survival and mate choice [2,3]. Individuals vary strongly in the expression of these traits due to genetic differences, differences in current conditions and conditions experienced during early development, such as the availability and/or quality of available food [4]. Especially in fast developing organisms, as for example in altricial songbirds, early nutritional stress causes immediate and long lasting effects on phenotypic development and fitness [5,6]. Early nutritional conditions have striking effects on biometry [7-9], longevity and reproductive success [2,10] as well as learning performance [11,12]. Also the development of personality as measured by exploration as one of the key operational measures of personality is affected by early dietary conditions [13-15] as well as early social conditions [16]. Such differences in exploration can have fitness relevant consequences in foraging or survival as well as consequences for mate choice and thus reproduction [17-19]. The early nutritional conditions are likely to specifically affect the development of secondary sexual characters as these usually are costly to produce. In species with female mate choice, females usually attend to such male sexually selected ornaments that reflect aspects of male quality [20]. Indeed, several studies on songbirds have shown that the expression of such sexual ornaments can be influenced by early nutritional stress which can cause a reduction of song attractiveness [21-26] and lead to less attractive visual ornamentation [27-29].

Yet, for some traits individuals might be able to compensate later on in life, for instance when nutritional conditions improve, and then benefit from such compensation. Indeed, songbirds that have experienced early developmental stress have been shown to compensate later in life in some traits like body mass [30-32], but not in others such as tarsus length[33]. Such compensations can carry costs and it may lead to trade-off decisions affecting other traits[5]. Studies in zebra finches (*Taeniopygia guttata*) have shown that compensation for body mass leads to a reduced cognitive performance [31], an elevated metabolic rate [30] and to an altered exploration behaviour [32].

If individuals compensate later in life for an initially poor expression of traits, they need to decide when to do so. For some traits it may pay to compensate as early as possible while for other traits compensation might better be delayed. Because sexually selected signals have a strong impact on finding a mate and thus fitness, selection indeed should favour individuals that can compensate early for initially poor expression of these traits. Birds are comparatively short lived and have limited opportunities to reproduce, so that they are likely to benefit by compensating first for deficits in the expression of sexual ornaments, providing that costs of low expression in other traits, such as reduced body mass, are low. Zebra finches have been a key model in studies on the effects of early developmental stress [34] as they mature relatively fast, are subjected to high mortality due to predation [35] and breed opportunistically [35,36]. Moreover, several studies have shown that male song [22-24] and female preference to male song [26] as well assexually selected plumage ornaments [29] are affected by early nutritional stress, but a study manipulating brood sizes did not find any effect using subjective visual scoring of the cheek patches [37]. In such a fast developing species with limited breeding opportunities, one would expect that selection will favour early mating and use of the first breeding opportunities. Investing in ornaments as early as possible then becomes important and males who prioritize compensating in initially low expression of ornaments will increase early attractiveness leading to a potentially higher mating success[38].

To test if males indeed are affected by early developmental stress in their expression of visual ornaments but compensate for them when conditions improve, we reared zebra finches under different nutritional conditions and analysed the expression of plumage ornaments at different stages during adolescence [32]. The subjects analysed here have been shown already to compensate in body mass at adulthood at the age of around 170 days [32]. This compensation in body mass was linked to altered exploration behaviour at this age [32]. We here thus examined whether prior to this body mass compensation (i) early poorer nutritional conditions have negative effects on the development of male cheek patches, one of the sexual plumage ornaments, and (ii) whether any initially lower expression in cheek patch size is compensated already at sexual maturation at day 65 of life. At this time zebra finch males’ spermatogenesis starts [35,39] and at a similar time pair formation begins, if a partner is available [35,40]. Furthermore, (iii) we examined whether or not activity and exploration behaviour is affected by early conditions [14-16] and compensation in cheek patch size. We expected early nutritional conditions to affect the growth of initial cheek patches [29] but that these effects would be compensated for at sexual maturation (day 65) [41].

## Results

## Cheek patches

**Fig. 1:**
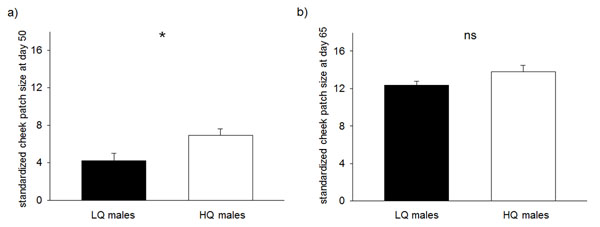
Mean male cheek patch size (±S.E.) of males reared in low quality (LQ; indicated by black bars; N=31) and high quality (HQ; indicated by white bars; N=29) early nutritional conditions at the age of a) 50 days post hatching and b) 65 days post hatching. At day 50 males from both treatments differed significantly in cheek patch size (LME day 50: factor nutritional treatment, p=0.046), whereas at day 65 this differences had been compensated by the low quality males (LME day 65: factor nutritional treatment, p=0.12). See text for details.

Male cheek patches at day 35 started to be expressed in only six (four HQ and two LQ) of the 60 males and thus were not directly compared with respect to the nutritional treatments. Male cheek patch size at day 50 was significantly affected by the early nutritional treatment with HQ males having larger cheek patches than LQ males (LME: nutritional treatment, F_1,35_=4.26, p=0.046), whereas neither the fathers’ cheek patch size (F_1,25_=0.12, p=0.73) nor the body mass at day 50 (F_1,21_=1.38, p=0.25) had a significant effect (Fig. 1a). Cheek patch growth from day 35-50 tended to be affected by early nutritional treatment, with HQ males tending to have a higher cheek patch growth than LQ males (LME: nutritional treatment F_1,36_=3.68, p=0.063). However at the age of 65 days, this difference had disappeared and there was no significant difference in cheek patch size between HQ and LQ males (LME: nutritional treatment, F_1,35_=2.51, p=0.12; fathers’ cheek patch size, F_1,35_=2.08, p=0.16; body mass at day 65, F_1,21_=0.53, p=0.48; Fig. 1b). The cheek patch growth from day 50-65 was affected mainly by the cheek patch size at day 50 rather than by the nutritional treatment itself (LME: nutritional treatment, F_1,36_=1.40, p=0.24; cheek patch size at day 50, F_1,21_=54.49, p<0.001); birds with larger cheek patches at 50 days showed less further cheek patch growth than males with smaller cheek patches at that age.

Cheek patches of adult males at day 280 were no longer different in size from each other with respect to early treatments (LME: nutritional treatment, F_1,34_=1.30, p=0.26). However, they tended to be positively linked to the paternal cheek patch size(F_1,34_=4.06, p=0.052), indicating a potentially heritable component of cheek patch size. Body mass at day 280 was again not affecting check patch size (F_1,21_=1.45, p=0.24). At day 280 HQ males had an average standardized cheek patch size of 15.30 ± 0.39 SE and LQ males of 15.97 ± 0.38 SE.

## Activity and exploration behaviour of adolescent males

**Fig. 2:**
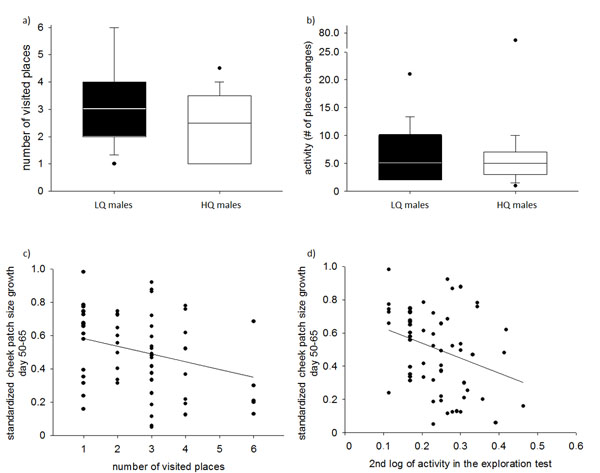
Males performance after day 65 in an exploration task. Males of early low quality (LQ; indicated by black bars; N=31) and early high quality (HQ; indicated by white bars; N=29) treatment did neither differ in a) the number of visited places nor b) in their activity in the test (panels a) and b) indicate the median, quartiles and outliers, i.e., data points that lie outside the 10th and 90th percentiles). However, compensatory cheek patch growth (from day 50-65) was negatively linked with the c) number of visited places and the d) total activity in the exploration test. See text for details.

The activity (number of place changes) in the test aviary was positively related to the body mass at day 65, i.e. heavier birds were more active. Cheek patch growth from day 50-65 was negatively correlated with activity; males having a higher cheek growth in that period were less active. Nutritional treatment had no direct significant effect on activity (LME: cheek patch growth, day 50-65, F_1,20_=5.22, p=0.033; body mass day 65, F_1,20_=7.00, p=0.015; nutritional treatment, F_1,36_=0.05, p=0.83; Fig 2b, d). The exploration behaviour, measured as the number of visited places, was correlated with activity (N=60, r_S_=0.74, p<0.0001). Furthermore exploration behaviour was negatively affected by cheek patch growth day 50-65, but, like activity, not by the nutritional treatment itself (GLMM: cheek patch growth, day 50-65, Z=-2.025, p=0.043; nutritional treatment, Z=-1.298, p=0.19; Fig. 2a,c).

## Discussion

We here show that zebra finch males which were raised under poorer nutritional conditions initially expressed smaller cheek patches at day 50 post-hatching but then compensated in cheek patch size already at 65 days, when becoming sexually mature. We further show that the compensatory growth in cheek patch during adolescence was negatively correlated with activity and exploration behaviour in a novel environment. These results suggest that not all individual traits are compensated with the same ontogenetic priority. The same zebra finches had compensated for reduced body mass much later, at adulthood at an age of around 170 days [32]. Thus, males appear to have invested until sexual maturation more resources in the growth of a sexually selected ornament, i.e., the cheek patch rather than in body mass. Such a strategy might well be adaptive as, with sexual maturation, males would benefit in finding a mate for reproduction as soon as possible. As zebra finches life in an unpredictable breeding environment and adjust breeding opportunistically to the weather conditions [35], missing a breeding opportunity is likely to have a negative impact on fitness. Therefore, males should benefit from being mature as well as attractive at the right time. Being late in cheek patch development thus could lead to disadvantages in mate choice [29].

This finding that deficits in secondary sexual ornaments are compensated until sexual maturation are in line with the predictions derived from a state-dependent dynamic optimization model that aimed to explore how resources during compensation were allocated to either soma or sexually ornaments [41]. However, Lindström et al.[41] also argued that a complete compensation in expected fitness is not very likely, as compensation in some traits will also have costs paid in another trait [e.g. [5], [7], [30] - [32]]. Our findings that young males gave priority to compensate in plumage attractiveness over body mass at this developmental stage indeed suggests that early compensation in one trait is paid by delayed compensation in another trait. Furthermore, we expected to find costs linked to the compensation in cheek patch size. Thus, we specifically tested whether activity (movements) as well as exploration behaviour (diversity of places visited) in an unfamiliar environment were linked to this compensation. Our finding that heavier birds who needed to compensate less for low body mass were more active is in line with the underlying expectation.

We found that the cheek patch growth in the time from day 50-65, i.e., the period during which the initial deficit in cheek patch size disappeared, was negatively correlated with activity and exploration behaviour. This reduced activity and exploration behaviour can have several potential disadvantages. When dispersing from the natal breeding site or colony [35,42], individuals are faced with new environments, where lower exploration behaviour can lead to more time in familiarization with the environment and fewer social contacts that may affect finding a suitable partner and time delays to reproduce. Being less explorative might also be interpreted as a positive feature of these individuals, as they might be exposed to less social competition, are possibly more thorough and learn faster [43,44] and save energy. However, once periods of starvation occur, a lower exploration behaviour would reduce the probability to find new food patches [14]. Lower foraging success during feeding offspring then might again trigger early nutritional stress in offspring, leading to non genetic trans-generational effects [33,45]. Neither activity nor exploration behaviour were directly affected by the early nutritional treatments but were negatively correlated to the compensatory cheek patch growth [46]. This is in line with other studies that reported effects on compensatory growth in the absence of direct effects of the nutritional treatments [31,32]. As suggested earlier [32], this underlines the importance to consider the effects of early nutritional conditions and subsequent compensatory growth separately, as both effects appear in different developmental phases and thus may require and trigger different mechanisms.

The idea that trade-offs in resource allocation are most likely leading to constraints in growing the cheek patch or body massis supported by various studies on moult [27,47]. It would also be interesting to measure the underlying physiological costs of cheek patch growth in future studies. Moreover, zebra finches reared initially on an intermediate diet but received high or low quality nutritional conditions during adolescence, from day 35-65 showed no differences in any biometric trait, but significant effects of nutrition during adolescence on cheek patch size [29]. Males with smaller cheek patches then were also less attractive in a mate choice test [29]. That study, together with the present results, suggests that for the development of an attractive cheek patch, the time after the independence is most important [35]. During this time early deficits can be compensated but when males experience poor conditions in this period compensation is delayed. Other important sexually selected male traits, in contrast, such as the song which is learnt early in life, are less flexible and remain a signal of nutritional stress experienced in early life [22,25,26,47].

Another interesting aspect we found post-hoc, is that the father's cheek patch size was not related to their sons’ cheeks during the early measurements (day 35,50,65). However, at adulthood (day 280) there was a tendency that fathers and sons cheek patch sizes were positively linked. This might indicate a potentially heritable component of cheek patch sizes and that environmental factors are more affecting the cheek patch growth than genetic factors.

Taken together, we show that males raised under early poor nutritional conditions compensated in cheek patch size already until sexual maturation for a bad start in life. This compensation in cheek patch size is likely to benefit male attractiveness but this compensation was related to lower exploration behaviour which may have fitness consequences as well [48]. These findings provide supporting evidence that compensatory priorities seem to exist and that not all deficits from a bad start are caught-up at once. Resource allocation in compensatory growth patterns should always maximise potential fitness at the current developmental stage.

## Methods

Subjects were 60 male zebra finches of wild Australian origin, which were about the F9 generation in the Bielefeld stock. In a breeding experiment, pairs of unrelated zebra finches were allowed to raise offspring under either high or low quality nutritional treatment conditions, and birds were followed over their development for growth and behavioural pattern [11,32,49]. All pairs received high quality food (see below) until the start of the nutritional treatments. Nutritional treatments started at day three post hatching of the oldest chick in a nest and ended at day 35, when chicks reached nutritional independence [35]. Thereafter all subjects received a diet of intermediate quality [49]. In the low quality (LQ) nutritional treatment subjects received standard seed food (a mixture of yellow millet, red millet, canary seed, and yellow panicum) *ad libitum* and only once a week additional egg food (egg food tropical finches, CéDé, Evergern, Belgium). In the high quality (HQ) nutritional treatment subjects received standard seed food *ad libitum* and daily protein rich egg food and germinated seeds (i.e. germinated seed food), as well as three times a week greens (chickweed, *Stellaria media*). Subjects from both treatments received daily fresh water which was twice a week supplemented with additional vitamins. After day 35 all subjects were housed until day 65 in song tutor groups (cage size 81 x 48 x 61 cm) with six to eight other juveniles of both sexes and an unrelated adult zebra finch pair and received a diet of intermediate quality. This diet consisted of *ad libitum* standard seed food, daily germinated seeds and three times a week egg. From day 65 on subjects were housed in mixed-sexed groups of three to four individuals in cages (83 × 30 × 39 cm). The breeding experiment and the consequences of early nutritional treatment on growth, behaviour and compensation in body mass of these birds have been described elsewhere [32,33,49].

## Cheek patch size

On days 35, 50 and 65 during maturation and at day 280at adulthood and after compensatory growth on body mass (see [32]) we took digital portrait photographs [29] from both sides of the males’ head using a Canon Power Shot S5IS digital camera. Photographs were taken from a standardized distance of 50 cm. From the digital photographs we calculated the male's standardized cheek patch sizes as the mean of the cheek size of the left and the right side. Standardized cheek patch size was calculated as the number of pixel of the cheek area divided by the number of pixels of the eye area on the pictures (modified after [50]; following [29]). Digital pictures were analysed with Adobe Photoshop 7.0. Each cheek patch area and eye area were measured three times and the average was used for further analysis. The repeated measures on the same patch correlated highly significantly with each other (all r_P_>0.974, p<0.00001).

We calculated cheek patch size growth rates as daily gain of standardized size (score/days) for the period after the nutritional treatments day 35-50 and for the compensatory period day 50-65. All fathers had also been photographed and their pictures analysed directly prior breeding started. Fathers had an average age of 787 days ± 387 days (S.D.).

## Test for exploration behaviour

Males were tested for their spontaneous exploration behaviour once in a novel environment after reaching day 65. At testing, subjects had on average an age of 84.9 days (± 17.1 SD). Subjects were, without prior food deprivation, transferred from their home cages to the unfamiliar experimental room and were tested there individually, without any visual or acoustical contact to conspecifics. Experiments were started from a start-box (20 x 20 x 20 cm) where subjects were allowed to calm down 5 min before testing. The novel environment was an arena (2.75 x 1.85 x 1.85m) exhibiting ten different locations to perch. During the tests no food was provided at any location in the arena. The start box was opened using a string from outside the experimental room, where the observer was behind an one-way window. After subjects had left the start box (average start-box time 177s ± 133 SD) and entered the arena, subject's exploration behaviour (perch changes) was measured for 10 min. We recorded (i) the number of visited places, i.e. how many different perching facilities in the novel environment were visited by an individual and (ii) the activity, i.e. the number of place changes in the novel environment.

## Biometry

Biometric measures were taken at different ages and have been reported elsewhere [32,33,49]. To summarize them briefly, body weight at hatching (day 0) was not different between the birds of the prospective nutritional treatments [49]. Nutritional treatment affected body mass, tarsus length and growth, i.e.,birds raised under LQ conditions were significant lighter during and at the end of the nutritional treatments at day 35 [49]. However, these effects on body mass lasted until the birds reached adulthood and were compensated by the age of about 170 days [32] and then remained not different during later life [32]. Tarsus length remained affected lifelong from early treatments [33].

## Statistical analysis

The measures were analysed in linear mixed effect models (LME) for the different ages. Cheek patch sizes were analysed using LMEs with nutritional treatment, body mass at the respective age, fathers’ cheek patch size and their two-way interactions as factors, and natal cage as random factor in the initial models. For the models analysing cheek patch size the factors fathers’ cheek patch size as well as body mass at the respective age remained in the final model to control for their effects on cheek size. The two cheek patch size growth rates were analysed using LMEs with nutritional treatment, cheek patch size at the beginning of the respective period, and fathers’ cheek patch size considered as factors and natal cage as a random factor.

Activity and number of visited places in the exploration test were analysed in mixed models (LME / GLMM with Poisson distribution) with nutritional treatment, body mass at day 65, cheek patch size growth day 35-50, cheek patch size growth day 50-65, exact age at testing with all two-way interactions as factors and natal cage as random factor. Residuals were tested for normal distribution using the Lilliefors Kolmogorov-Smirnov test. Some parameters were once or 2nd log (x+1) transformed to reach this criterion [51]. Non significant terms were step wise excluded in a backward selection from the initial model to reach the final, most parsimonious model. Nutritional treatment remained in the final models to control for the effects. Analyses were conducted using R 2.9.0, using the packages nlme and lme4 and SPSS 19.

## Ethical note

The experiments were carried out according to the German Lawsfor experimentation with animals. Breeding and housing of birds was conducted with permission of the Veterinäramt Bielefeld, Germany (permit number 530.42 1630-1). After the experiments all birds remained in the laboratory stock of Bielefeld University.

## Authors’ contributions

Conceived and designed the experiments: ETK, MN. Performed the experiments: ETK. Analyzed the data and wrote the paper: ETK MN.
